# Barriers and Facilitators for Information Exchange during Over-The-Counter Consultations in Community Pharmacy: A Focus Group Study

**DOI:** 10.3390/pharmacy5040065

**Published:** 2017-12-06

**Authors:** Liza J Seubert, Kerry Whitelaw, Fabienne Boeni, Laetitia Hattingh, Margaret C Watson, Rhonda M Clifford

**Affiliations:** 1 Division of Pharmacy, The University of Western Australia, M315, 35 Stirling Hwy, Crawley, WA 6009, Australia; kerry.whitelaw@uwa.edu.au (K.W.); rhonda.clifford@uwa.edu.au (R.M.C.); 2 Department of Pharmaceutical Sciences, Pharmaceutical Care Research Group, University of Basel, Klingelbergstrasse 50, CH-4056 Basel, Switzerland; fabienne.boeni@unibas.ch; 3 School of Pharmacy and Pharmacology, Griffith University, Gold Coast Campus, Southport, QLD 4222, Australia; L.Hattingh@curtin.edu.au; 4 Department of Pharmacy and Pharmacology, University of Bath, 5W 3.33, Claverton Down, Bath BA2 7AY, UK; m.c.watson@bath.ac.uk

**Keywords:** communication, nonprescription drugs, over-the-counter drugs, pharmacists, pharmacy, focus group

## Abstract

Consumers are confident managing minor ailments through self-care, often self-medicating from a range of over-the-counter (OTC) medicines available from community pharmacies. To minimise risks, pharmacy personnel endeavour to engage in a consultation when consumers present with OTC enquiries however they find consumers resistant. The aim was to determine stakeholder perspectives regarding barriers and facilitators for information exchange during OTC consultations in community pharmacies and to understand the elicited themes in behavioural terms. Focus groups were undertaken with community pharmacist, pharmacy assistant and consumer participants. Independent duplicate analysis of transcription data was conducted using inductive and framework methods. Eight focus groups involving 60 participants were conducted. Themes that emerged indicated consumers did not understand pharmacists’ professional role, they were less likely to exchange information if asking for a specific product than if asking about symptom treatment, and they wanted privacy. Consumers were confident to self-diagnose and did not understand OTC medicine risks. Pharmacy personnel felt a duty of care to ensure consumer safety, and that with experience communication skills developed to better engage consumers in consultations. They also identified the need for privacy. Consumers need education about community pharmacists’ role and responsibilities to motivate them to engage in OTC consultations. They also require privacy when doing so.

## 1. Introduction

Many consumers are confident in the management of their minor ailments through self-care and self-medication [1,2]. This is facilitated by an interest in personal health, accessible health information through the internet and access to a variety of over-the-counter (OTC) medicines available without prescription [3]. Self-care and self-medication of minor ailments have benefits such as consumer empowerment, convenience and reducing healthcare costs associated with clinic or general practice visits [2,4]. Possible risks must also be considered such as the potential for incorrect self-diagnosis and subsequent delay in accessing appropriate treatment, interactions with concomitant medicines, and inappropriate use of medicines [2,4].

Pharmacists and other pharmacy personnel are readily accessible in community pharmacies and as such ideally placed to support consumers with self-care. The World Health Organisation describes several functions of pharmacists involved with self-care requests [5], the primary function being a ‘communicator’. The pharmacist engages with a consumer to obtain information relevant to the enquiry and provides information to assist the consumer to select appropriate medication or refer the consumer to another health professional when necessary. Similarly, professional organisations, recognise the pharmacist’s role in the provision of OTC medicines and the supervision of pharmacy personnel in the supply of these products [6].

Globally, countries are increasing access to medicines which were previously only available as prescription medicines to enable self-care and reduce national drug budgets [7,8,9]. The range of OTC medicines is available to consumers with varying levels of pharmacist involvement required prior to supply, depending on the classification and country [10,11,12,13].

Pharmacists are entrusted with considerable responsibility to facilitate the appropriate use of OTC medicines as well as providing many other professional services such as dispensing of prescription medicines, medication reviews, screening and risk assessment, and compounding [6]. As pharmacists adopt more extended roles, they rely on other pharmacy personnel, such as pharmacy assistants, to provide certain services.

OTC requests can be complex and interventions to improve OTC consultations and support consumers to engage in self-care have been implemented in the community pharmacy setting with variable success [14,15,16,17,18]. Pharmacists and pharmacy personnel report difficulties in engaging consumers in dialogue, particularly when consultation involves a request for a medicine by name (hereafter referred to as a product based request) [14,19,20].The reasons for this lack of engagement require exploration and it is reasonable to suggest that there is not one simple reason, but rather a range of factors that interact.

**Figure 1:**
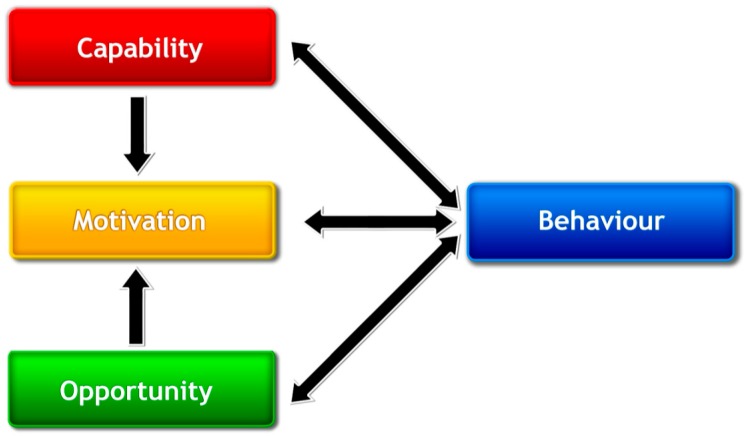
The COM-B system—a framework for understanding behaviour [24].

Models and theories can provide greater understanding of the determinants of different behaviours such as consumer engagement in OTC consultations. The development of the Theoretical Domains Framework (TDF) identified the main factors that influence behaviours of healthcare professionals (Supplementary file 1) [21,22]. The Behaviour Change Wheel [23] is a synthesis of a number of behaviour change frameworks with the COM-B model of behaviour at its centre (Supplementary file 2). The COM-B model (Figure 1) recognises that behaviour is a result of the interacting components of capability, opportunity and motivation (COM), and that behaviour (B) in turn may also influence capability, opportunity and motivation [23,24]. Cane et al. mapped the COM-B system to the TDF domains when validating the TDF [22]. Mapping the TDF to COM-B can assist researchers to identify the target for interventions that aim to change behaviour. While it is recognised that the use of behavioural theory may assist to understand behaviours, few community pharmacy-specific interventions utilise behavioural theory to develop or report interventions [25].

The aim of this study was to determine stakeholder perspectives regarding barriers and facilitators for information exchange during OTC consultations in community pharmacies. A secondary aim was to understand the elicited barriers and facilitators in behavioural terms.

## 2. Materials and Methods

The data collected from this research will be used to inform the development of an intervention to enhance OTC consultations between pharmacy personnel and consumers in community pharmacies.

### 2.1. Study Design

A series of focus group discussions were undertaken to elicit a broad range of perspectives from key stakeholders to enable a deeper understanding of the topic. The main focus of these discussions was to explore the barriers and facilitators to the exchange of information between consumers and pharmacy personnel when consumers present in a community pharmacy with a request for a specific product (product based request) or assistance with a symptom (symptom based request)—hereafter referred to as an OTC consultation.

Approval for the conduct of this study was obtained from the Human Research Ethics Office at the University of Western Australia (RA/4/1/5298).

### 2.2. Participants

The key stakeholders of OTC consultations are consumers, pharmacists and pharmacy assistants. As such, these were the participants recruited to this study using purposive sampling to recruit 4–12 participants for each focus group. Participants were recruited from Perth, Western Australia. Pharmacist participants were recruited by email invitation from a member of The University of Western Australia (UWA) pharmacy practice teaching team to her contacts. This included more than 40 community pharmacists with whom Master of Pharmacy students were placed for practicums and pharmacists who taught into the course. Pharmacy assistant participants were recruited by pharmacist participants and the UWA pharmacy practice teaching team. Inclusion criteria for pharmacy personnel were that they had previous or current experience working in community pharmacy. Consumers were recruited via poster advertisements at UWA and within one Perth metropolitan community pharmacy with a UWA association during April and May of 2012 and again in April and May of 2013. Consumer participants were provided a $20 voucher for participation.

### 2.3. Focus Group Design

Focus group discussions were conducted by focus group moderators (LS, LG, HA). LS, an experienced focus group moderator, conducted training for LG and HA, Master of Pharmacy student researchers. The training was in the format of the provision of information about focus group facilitation, an observation of LS conducting a focus group, a pilot focus group consisting of Master of Pharmacy students as participants for practise (results not included in presented data), and feedback on technique. Each focus group commenced by discussing the focus group rules with a general outline of the project, including specific aims. During this time, participants were provided with participant information sheets and a demographic form. Consent forms were subsequently completed for both participation in and audio-recording of the focus group discussion. Consent forms were identical for all participant groups. Participant Information Sheets also had the same content, substituting lay person language for a few words in the consumer document. The focus group moderators followed a thematic interview guide which was adapted for the different participant groups (Table 1). The interview guide was developed by members of the research team (LS, RC, KW) with extensive community pharmacy experience and informed by a search of the literature for factors that influence OTC consultations [14,15,18,20,26,27,28,29,30,31,32,33,34,35]. The focus group discussions were conducted systematically beginning with a general discussion of participants’ perceptions of asking/answering health-related questions during OTC consultations, followed by a more specific discussion of barriers and facilitators of the information exchange process.

### 2.4. Data Collection and Analysis

During the focus group discussions, a second researcher took notes on the participant responses. The discussions were also audio-recorded and then transcribed *verbatim*. A general inductive approach [36] was used for the analysis of the transcripts and was conducted independently by two researchers (LS, DA—a researcher with focus group expertise). Categories of key themes from the content of the discussions emerged into which the data was coded. Coding disagreements were discussed until consensus was reached.

The framework method of analysis [37] was also applied independently by two researchers (LS, KW) who coded data to the COM-B model and Theoretical Domains Framework (TDF) [22,23]. Coding disagreements were discussed until consensus was reached. 

## 3. Results

Eight focus groups were held during 2012 and 2013 in Western Australia (Table 2). A total of 60 people participated in the focus group discussions (33 pharmacy personnel, 27 consumers). Statistics from the Pharmacy Board of Australia reports that in June 2013, 61% of Western Australian registered pharmacists were female [38]. Each focus group was approximately one hour in duration.

**Figure 2:**
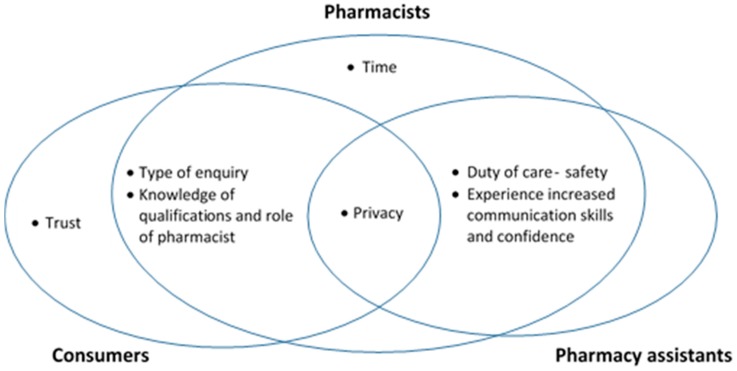
Thematic map of focus group themes and overlap.

Figure 2 illustrates the overlapping themes that emerged from the focus group discussions when participants were asked about barriers and facilitators for asking/answering health related questions during OTC consultations.

The results of the TDF/COM-B analysis are summarised in Table 3 [23]. There was greater coding density for the Capability COM-B component from pharmacist participants than consumer participants which related to pharmacists’ perception that consumer’s lacked knowledge about the role of the pharmacist and also lacked knowledge about the potential risks with OTC medicines. Pharmacists also recognised the development of interpersonal (consultation) skills with experience. Environmental context and resources coding occurred frequently from the Opportunity domain and from the Motivation domain social/professional role and identity, belief about capabilities, and belief about consequences occurred frequently. Quotes are provided to illustrate the themes from both coding analyses.

COM-B: Capability. TDF: Cognitive and interpersonal skills

In the opening discussion of the focus group, pharmacy personnel reflected that as their communication skills developed with experience so too did their confidence and ability to develop a rapport with consumers and this facilitated asking consumers questions.

“I think to build a rapport with customers is important. The first contact [with the consumer] is quite important. If you can make a little difference on the first contact they will probably come back again and want to spend more time in the pharmacy. And that’s the professionally rewarding bit.” **Pharmacists’ focus group 2, Participant 3** (hereafter quotes are labelled in the format **Pharmacist 2, Participant 3**)

“I guess that’s where the skill comes in…using a different approach with different people.” **Pharmacist 3, Participant 8**

“It’s [communication] a skill set that does take years of experience to develop.” **Pharmacist 3, Participant 2**

“I think after working in pharmacy for five years … you get used to it. Initially it was difficult to develop that relationship to ask personal questions …but with time you become a bit more comfortable.” **Pharmacist 4, Participant 4**

“[it helps to read] the body language of customers when you ask them the first question.” **Pharmacy Assistant 1, Participant 1**

COM-B: Capability. TDF: Knowledge

Pharmacist participants expressed that they felt consumers’ lack of knowledge about the qualifications, obligations and role of the pharmacist made consumers resistant to their questions.

“I think people probably underestimate the knowledge that pharmacists have.” **Pharmacists 2, Participant 2**

*“… people just don’t know what we do. They don’t know what our skill base is and how we can help them*.” ***Pharmacists 2****, **Participant 1***

*“… it really does depend on how they approach you and what they think you can offer.… most of the time people are very happy to offer some information but, you know, sometimes people are like: what right do you have to be asking me questions?*” ***Pharmacists 3****, **Participant 3***

To overcome this barrier pharmacists suggested publicising the role of the pharmacist.

“I think we need a massive PR [Public Relations] exercise on what pharmacists actually do, because people don’t know what we do … they don’t know what our skill base is and how we can help them” **Pharmacists 2**, **Participant 1**

“… a massive public [relations exercise] on what the schedules are and what the role of the pharmacist is and that it isn’t a retail transaction.” **Pharmacists 3, Participant 6**

This was echoed by participants from consumer focus groups who indicated that they lacked an understanding of the role of the pharmacist in OTC symptom or product enquiries. 

*“I’m not often sure what I can ask the pharmacist.” **Consumer 2***, ***Participant 4***

*“I wouldn’t expect them to just hand it [OTC product] over … there would be a couple of corporate [standard] questions I would expect. Are you allergic, for instance. Those questions would be adequate, not about the actual problem. Not about the headache* etc.*” **Consumer 1, Participant 2***

“They don’t have to ask for everyday things like Panadol^®^ [paracetamol] and Nurofen^®^ [ibuprofen] … because it’s an everyday drug for a reason. Because it’s OK for most people to take.” **Consumer 2, Participant 3**

COM-B: Motivation. TDF: Social and professional role and identity. TDF: Beliefs about consequences

However, some consumer participants stated they thought that pharmacists had a duty of care to ensure the safety of the consumer.

“I think pharmacists have a responsibility to ask questions because they can provide professional advice for certain diseases you may have. I think it’s necessary.” **Consumer 1, Participant 4**

*“The most important question with medications is [about] interactions. There are just so many of them” **Consumer 3, Participant 2** “and not everything is compatible and you’re not aware that it would be really wrong to take them with something else that you’re taking.”* added ***Consumer 3, Participant 7***

Pharmacy assistant and pharmacist participants expressed an obligation to ensure the safety of the consumer.

“It’s a duty to ask them [health questions] to make sure we know what’s going on with their life instead of giving them something that could kill them.” **Pharmacy Assistant 1**, **Participant 1**

“If somebody says ‘I’ve had this before. Why are you asking me these questions?’ If I say to them ‘Well, because if this happens … you can end up in this scenario and that’s why I’m asking. Just to keep you safe.’” **Pharmacist 3, Participant 7**

COM-B: Motivation. TDF: Social and professional role and identity. TDF: Beliefs about capabilities

Pharmacy assistants expressed confidence in asking the pharmacist for assistance with OTC enquiries and that consumers may provide different information to the pharmacist.

“I think it’s important to make that decision to involve the pharmacist. People actually like that. They like to speak to the pharmacist … they really appreciate it when you involve the pharmacist.” **Pharmacy Assistant 1, Participant 4**

“It’s amazing how their information changes from talking to us and talking to the pharmacist. It’s like ‘you never said that! I asked you that question and you said no, and now you’re telling the pharmacist something different!’” **Pharmacy Assistant 1**, **Participant 2**

A consumer participant stated willingness to provide health information to the pharmacist but not the pharmacy assistant. Other consumers noted that they could not identify who was a pharmacist and who was a pharmacy assistant (coded to COM-B: Opportunity. TDF: Environmental context and resources) 

“I guess it depends on who I am talking to. I’d be comfortable talking to the pharmacist … but if I’m talking to just an assistant … I wouldn’t be as comfortable disclosing that information.” **Consumer 1, Participant 9**

“You don’t know if the person you’re speaking to is the actual pharmacist or a staff person and you don’t know how much they’ve studied.” **Consumer 2, Participant 12**

“… you can’t tell the difference between pharmacist and assistants—there is an issue there I think.” **Pharmacist 1, Participant 4**

COM-B: Motivation. TDF: Beliefs about capabilities. TDF: Beliefs about consequences

Pharmacist and consumer participants noted more willingness to exchange information with symptom based requests as opposed to product based requests. Pharmacist and consumer participants noted that the perceived risk, nature, or sensitivity of the problem also impacted the willingness of consumers to provide information. If a consumer asked for a product by name they were confident with their ability to manage the issue. However, if the request was for treatment of a symptom they were less confident.

In this context the need for privacy was universally raised. (Privacy is coded to COM-B: Opportunity. TDF: Environmental context and resources)

“For cold and flu I’m perfectly happy to give over that information. But if it was something I deemed to be more personal …. I would be very resistant [to providing information] because I guess there is no privacy.” **Consumer 1, Participant 2**

“For things like a cold and everyday things I really don’t mind what questions they ask. But if it was a more personal thing it might be really specific and I’d be more reserved.” **Consumer 2, Participant 8**

“I find it more [challenging asking questions] if they are asking for a particular product, then they aren’t after the questions …. But if they come in with a problem then they want your help … then they are up for the questioning.” **Pharmacist 4, Participant 7**

“The other issue is privacy. I mean you ask these questions standing at a counter with other people around and it’s not necessarily appropriate [consumers] giving [their] medical history.” **Pharmacist 1, Participant 4**

“I think there’s a good chunk of consumers who consider pharmacy as a transactional source of goods that they may not even view as a medicine. In their eyes ‘I don’t need a script [prescription] for it so just give it to me.’” **Pharmacist 3, Participant 7**

COM-B: Opportunity. TDF: Environmental context and resources

Pharmacists expressed concern with the limited time they had available to interact with consumers.

“Time is definitely a barrier because of the nature of our business. People rush in and you’ve got to serve everybody.” **Pharmacist 1, Participant 1**

“There is a lot of pressure with people coming in and wanting to get their scripts in a timely manner [preventing time spent with OTC consumers].” **Pharmacist 4, Participant 5**

## 4. Discussion

This qualitative study on OTC consultation behaviour in community pharmacies explored barriers and facilitators to engaging in information exchange. A wide range of views from different perspectives were elicited during the discussions with convergent opinions between pharmacy personnel and consumers. The findings highlight consumer expectation of minimal interaction with OTC enquiries primarily due to a lack of knowledge about the professional role and obligations of pharmacists and other pharmacy personnel.

Pharmacy personnel are charged with supporting consumers in the management of their OTC enquiries through minimising the risks that consumers may not associate with self-care and self-medication. Pharmacy personnel and consumers expressed the view that pharmacists have a duty of care to ensure the safety of consumers with OTC enquiries. However, consumers seemed to relate this duty to only ensuring the consumer was not allergic to a medicine supplied or that it did not interact with their other medicines. While consumers were more willing to exchange health information if their request was for a symptom than for a specific product/medicine, it was evident that many consumers had a poor knowledge of the professional role of the pharmacist in diagnosis and whole of patient care.

Consumers’ comments indicated an underestimation of the risks associated with taking OTC medicines and some were of the view that as these medicines were available without a prescription they had a right to obtain them without the need to engage in a discussion with the pharmacist. In behavioural terms, consumers are motivated to purchase an OTC medicine without consultation because they believe they have the capability to determine which medicine they require. This rise in health consumerism has been investigated by Hibbert et al. who found that many consumers were very confident in their ability for self-diagnosis and self-care of some conditions with purchased medicines [1]. Others have reported similar dis-interest of consumers to engage in a conversation about medicines [39], particularly when a specific product is requested as opposed to a request for treatment of a symptom [20,27,29].

A reluctance to engage in a discussion with OTC enquiries might also be related to the observation by all participant groups that consumers did not understand the role and responsibilities of pharmacists and other pharmacy personnel. The services offered by pharmacists have evolved from medicine supply to holistic patient care. It is broadly reported that consumers do not understand what services pharmacists are capable of providing [39,40]. To address this deficit in the knowledge TDF domain, campaigns have been implemented in many countries to improve the public knowledge of the pharmacist’s role. For example, there is a Pharmacy Awareness Month in Canada [41], Australia has an ‘Ask your Pharmacist’ campaign [42] promoting pharmacists as a trusted source of health services and advice, and a similar campaign is run in the UK—‘Dispensing Health’ [43].

Pharmacist and pharmacy assistant participants recognised the need to develop a rapport with consumers when they present with an OTC enquiry. To develop this capability they said they needed to have well developed communication skills and that the more experience they had the better they reported having the required skill. This perception has been investigated by Nguyen who studied the development of pharmacist-patient consultation interactions in novice pharmacists (pharmacists in their first year of practice) and how they become ‘expert’ [44]. She found that the development of competence over their first year of practice occurred through repeated participation in interactions with patients. Novice pharmacists developed the skills of a two-way interaction involving the dynamics of turn taking, topic management, action sequencing, linguistic forms and participation frameworks. Novice pharmacists used their experiences to adjust future interactions resulting in a gradual change towards ‘expertness’ [44].

Privacy is a factor that affects the behaviour of consumers during OTC consultations. In Western Australia, where the study was conducted, pharmacies are required by law to have an area where consultations cannot reasonably be expected to be overheard [45]. While the legislation falls short of requiring a consultation room, in the time since the focus groups were conducted, immunisation by pharmacists has been implemented in Australia and requires community pharmacies where immunisation is provided to have a private consultation area [46]. Although this may provide a private area for some services it is likely that the majority of OTC consultations will continue to occur at the counter. When designing pharmacy layouts planning needs to provide easily accessible areas for private consultations to occur out of the hearing of the general public to provide consumers with the opportunity to engage in a consultation [47].

A strength of this study was the input from key participants involved in OTC consultations. The external validity of the analyses is strengthened by the use of theoretical frameworks. A limitation is that similar numbers from each participant group were not achieved resulting in a greater number of consumer and pharmacist participants than pharmacy assistants which may not fully represent the views of pharmacy assistants. Other limitations common to focus group methodology include the inability to generalise the qualitative data, and that the data could have been misinterpreted. This limitation was addressed by duplicate independent coding of the data.

## 5. Conclusions

In this study, many issues relating to information exchange during OTC enquiries in community pharmacy were identified. There was considerable overlap with the barriers and facilitators identified between participant groups. Much work has been conducted to improve the knowledge and skills (Capability) of pharmacists and pharmacy assistants when responding to OTC enquiries in community pharmacies with varying degrees of success [14,20,48,49,50,51]. There is, however, little research where the consumer is the target of the intervention. This study highlighted the consumer perspective as well as perspectives from pharmacy personnel. Educating (Capability) consumers about the role of the pharmacist and the potential risks associated with medicines (Motivation) whether they are available with or without a prescription is one type of intervention which might address these communication barriers.

## Figures and Tables

**Table 1:** Thematic interview guide.

Participant Group	Main Themes	Support Questions
Pharmacist and pharmacy assistant	1. How do you feel about asking patients questions about their health?	- Do you think it is necessary? Why?
	2. What hinders patient assessment for over the counter enquiries?	- How does time affect asking questions? - Do you feel privacy is a factor? Why?
	3. What helps patient assessment for over the counter enquiries?	- How do you feel about taking a written patient history for primary care scenarios?
Consumers	1. How do you feel about being asked questions about your health by the pharmacist/pharmacy staff?	- Do you think it is necessary? Why?
	2. What closes the conversation about your health with the pharmacist/pharmacy staff?	- How does time affect asking questions? - Do you feel privacy is a factor? Why?
	3. What helps a conversation about your health with the pharmacist/pharmacy staff?	- How would you feel if the pharmacist took a written history from you for an over the counter enquiry?

**Table 2:** Focus group participant demographics.

Stakeholder Group	Pharmacists	Pharmacy Assistant	Consumer
Number of focus groups conducted	4	1	3
Participant numbers	28	5	27
Female %	71	80	85
Mean age, years (range)	35 (21–62)	33 (20–57)	35 (17–82)
Median years since registration (IQR)	6 (25)	8 ^#^ (11)	NA
Currently working:			
• Full time	16	1	3
• Part time	11	4	14
• Not working	1 *	0	10

* on maternity leave, ^#^ mean years working in pharmacy.

**Table 3:** COM-B and Theoretical Domains Framework (TDF) coding from focus group transcripts.

			Participants (n)		
**COM-B**	**TDF**		Pharmacist (28)	Pharmacyassistant (5)	Consumer (27)
**CAPABILITY**	**Physical**	**Physical skills**	-	-	-
	**Psychological**	**Knowledge**	✓✓✓✓	-	✓
		**Cognitive & interpersonal skills**	✓✓✓	✓	-
		**Memory, attention & decision processes**	✓✓	✓	✓
		**Behavioural regulation**	✓	✓	✓
**OPPORTUNITY**	**Social**	**Social influences**	✓	-	✓
	**Physical**	**Environmental context & resources**	✓✓✓✓	✓	✓✓✓
**MOTIVATION**	**Reflective**	**Social & professional role & identity**	✓✓✓✓	✓	✓✓✓
		**Belief about capabilities**	✓✓	✓	✓✓
		**Optimism**	✓	-	-
		**Belief about consequences**	✓✓✓	✓	✓✓
		**Intentions**	✓	✓	✓
		**Goals**	✓	-	-
	**Automatic**	**Reinforcement**	✓	-	-
		**Emotion**	✓	✓	✓

## Supplementary Materials

The following are available online at www.mdpi.com/2226-4787/5/4/65/s1. Supplementary file 1—Mapping of COM-B components to the Theoretical Domains Framework. Supplementary file 2—The COM-B system—a framework for understanding behaviour.
