# Supplementary material for: Barriers and Facilitators for Information Exchange during Over-The-Counter Consultations in Community Pharmacy: A Focus Group Study
Source: Pharmacy (Basel). 2017 Dec 6;5(4):65. doi: 10.3390/pharmacy5040065 (PMC5748546; doi:10.3390/pharmacy5040065)
Supplement: Supplementary file 1 [file pharmacy-05-00065-s001.pdf]

Supplementary file 1

Table 1. Mapping of COM-B components to the Theoretical Domains Framework[1]

COM-B component	Domain (definition)	Constructs
**CAPABILITY**		
**Physical**	**Physical skills**<br>(An ability or proficiency acquired through practice)	Skills<br>Skills development<br>Competence<br>Ability<br>Practice<br>Skill assessment
**Psychological**	**Knowledge**<br>(An awareness of the existence of something)	Knowledge (including knowledge of condition/scientific rationale)<br>Procedural knowledge<br>Knowledge of task environment
	**Cognitive & interpersonal skills**<br>(An ability or proficiency acquired through practice)	Skills<br>Skills development<br>Competence<br>Ability<br>Interpersonal skills<br>Practice<br>Skill assessment
	**Memory, attention & decision processes**<br>(The ability to retain information, focus selectively on aspects of the environment and choose between two or more alternatives)	Memory<br>Attention<br>Attention control<br>Decision making<br>Cognitive overload/tiredness
	**Behavioural regulation**<br>(Anything aimed at managing or changing objectively observed or measured actions)	Self-monitoring<br>Breaking habit<br>Action planning
**OPPORTUNITY**		
**Social**	**Social influences**<br>(Those interpersonal processes that can cause individuals to change their thoughts, feelings, or behaviours)	Social pressure<br>Social norms<br>Group conformity<br>Social comparisons
**Physical**	**Environmental context & resources**<br>(Any circumstance of a person's situation or environment that discourages or encourages the development of skills and abilities, independence, social competence and adaptive behaviour)	Environmental stressors<br>Resources/material resources<br>Organisational culture/climate<br>Salient events/critical incidents<br>Person × environment interaction<br>Barriers and facilitators
**MOTIVATION**		

Reflective	**Social & professional role & identity**<br>(A coherent set of behaviours and displayed personal qualities of an individual in a social or work setting)	Professional identity<br>Professional role<br>Social identity<br>Identity<br>Professional boundaries<br>Professional confidence<br>Group identity<br>Leadership<br>Organisational commitment
	**Belief about capabilities**<br>(Acceptance of the truth, reality or validity about an ability, talent or facility that a person can put to constructive use)	Self-confidence<br>Perceived competence<br>Self-efficacy<br>Perceived behavioural control<br>Beliefs<br>Self-esteem<br>Empowerment<br>Professional confidence
	**Optimism**<br>(The confidence that things will happen for the best or that desired goals will be attained)	Optimism<br>Pessimism<br>Unrealistic optimism<br>Identity
	**Belief about consequences**<br>(Acceptance of the truth, reality, or validity about outcomes of a behaviour in a given situation)	Beliefs<br>Outcome expectancies<br>Characteristics of outcome expectancies<br>Anticipated regret<br>Consequents
	**Intentions**<br>(A conscious decision to perform a behaviour or a resolve to act in a certain way)	Stability of intentions<br>Stages of change model<br>Transtheoretical model and stages of change
	**Goals**<br>(Mental representations of outcomes or end states that an individual wants to achieve)	Goals (distal/proximal)<br>Goal priority<br>Goal/target setting<br>Goals (autonomous/controlled)<br>Action planning<br>Implementation intention
Automatic	**Reinforcement**<br>(Increasing the probability of a response by arranging a dependent relationship, or contingency, between the response and a given stimulus)	Rewards (proximal/distal, valued/not valued, probable/improbable)<br>Incentives<br>Punishment<br>Consequents<br>Reinforcement<br>Contingencies<br>Sanctions

	**Emotion**<br>(A complex reaction pattern, involving experiential, behavioural, and physiological elements, by which the individual attempts to deal with a personally significant matter or event)	Fear<br>Anxiety<br>Affect<br>Stress<br>Depression<br>Positive/negative affect<br>Burn-out

Reference

1. Cane J; O'Connor D; Michie S. Validation of the theoretical domains framework for use in behaviour change and implementation research. *Implement Sci* **2012**, *7*.
