# Supplementary material for: Barriers and Facilitators for Information Exchange during Over-The-Counter Consultations in Community Pharmacy: A Focus Group Study
Source: Pharmacy (Basel). 2017 Dec 6;5(4):65. doi: 10.3390/pharmacy5040065 (PMC5748546; doi:10.3390/pharmacy5040065)
Supplement: Supplementary file 2 [file pharmacy-05-00065-s002.pdf]

**Supplementary file 2**

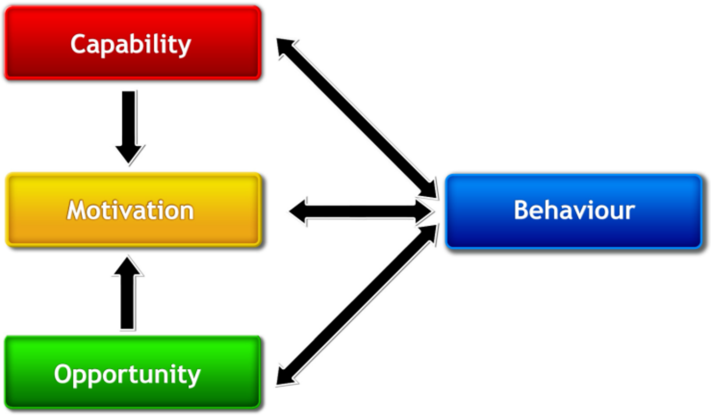


**Figure 1.** The COM-B system - a framework for understanding behaviour [1].

**Capability** - the individual's psychological and physical capacity to engage in the activity concerned. It includes having the necessary knowledge and skills.

**Motivation** - all the brain processes that energize and direct behaviour, not just goals and conscious decision-making. It includes habitual processes, emotional responding, as well as analytical decision-making.

**Opportunity** - all the factors that lie outside the individual that make the behaviour possible or prompt it.

**Reference**

1. Michie S; van Stralen MM; West R. The behaviour change wheel: A new method for characterising and designing behaviour change interventions. *Implement Sci* **2011**, *6*.
